# A tree-based scan statistic for zero-inflated count data in post-market drug safety surveillance

**DOI:** 10.1038/s41598-022-19998-5

**Published:** 2022-09-29

**Authors:** Goeun Park, Inkyung Jung

**Affiliations:** Division of Biostatistics, Department of Biomedical Systems Informatics, Yonsei University College of Medicine, 50-1 Yonsei-ro, Seodaemun-gu, Seoul, 03722 Korea

**Keywords:** Health care, Mathematics and computing

## Abstract

After new drugs enter the market, adverse events (AE) induced by their use must be tracked; rare AEs may not be detected during clinical trials. Some organizations have been collecting information on suspected drugs and AEs via a spontaneous reporting system to conduct post-market drug safety surveillance. These organizations use the information to detect a signal representing potential causality between drugs and AEs. The drug and AE data are often hierarchically structured. Accordingly, the tree-based scan statistic can be used as a statistical data mining method for signal detection. Most of the AE databases contain a large number of zero-count cells. Notably, not only an observational zero from the Poisson distribution, but also a true zero exists in zero-count cells. True zeros represent theoretically impossible observations or possible but unreported observations. The existing tree-based scan statistic assumes that all zeros are zero-valued observations from the Poisson distribution. Therefore, true zeros are not considered in the modeling, which can lead to bias in the inferences. In this study, we propose a tree-based scan statistic for zero-inflated count data in a hierarchical structure. According to our simulation study, in the presence of excess zeros, our proposed tree-based scan statistic provides better performance than the existing tree-based scan statistic. The two methods were illustrated using Korea Adverse Event Reporting System data from the Korea Institute of Drug Safety and Risk Management.

## Introduction

After new drugs enter the market, the adverse events (AE) induced by their use must be tracked because rare AEs may not be detected during clinical trials owing to short trial durations, limited sample sizes, or limited population representation. Once drugs are commercialized, they are used in different ways and by more people than those covered during clinical trials. Accordingly, drug safety must be monitored even after commercialization to identify AEs that may not have been identified previously^1–7^.

Drug and vaccine safety monitoring systems have traditionally been based on spontaneous reporting systems, such as the US Food and Drug Administration’s Adverse Event Reporting System (AERS), the US Vaccine Adverse Event Reporting System (VAERS), and VigiBase, the World Health Organization’s (WHO) global Individual Case Safety Reports database. AERS is a large database supporting the US Food and Drug Administration’s program for monitoring drug safety; VAERS helps monitor vaccine-related AEs and is maintained by the US Center for Disease Control and Prevention and the US Food and Drug Administration; and VigiBase is managed by the Uppsala Monitoring Centre (UMC) on behalf of the WHO. VigiBase receives individual case safety reports from 80 countries. In South Korea, the Korea Institute of Drug Safety and Risk Management provides information on AEs collected through the Korea Adverse Event Reporting System (KAERS) to the UMC. These spontaneous reporting systems play an important role in detecting AE signals in post-market drug safety surveillance^8,9^.

Disproportionality data mining methods have been used to analyze these databases to identify signs that certain drugs may be posing unrecognized safety hazards. Frequentist methods, such as the proportional reporting ratio^10^, relative odds ratio^11^, Yule’s test^12^, chi-squared test^13^, and likelihood ratio test (LRT)^14^, and Bayesian methods, including the Bayesian confidence propagating neural network^15^, multi-item gamma Poisson shrinker^16^, and simplified Bayes (sB) methods^15–19^ are often used to detect drugs with previously unrecognized AE^16,20–25^.

**Figure 1:**
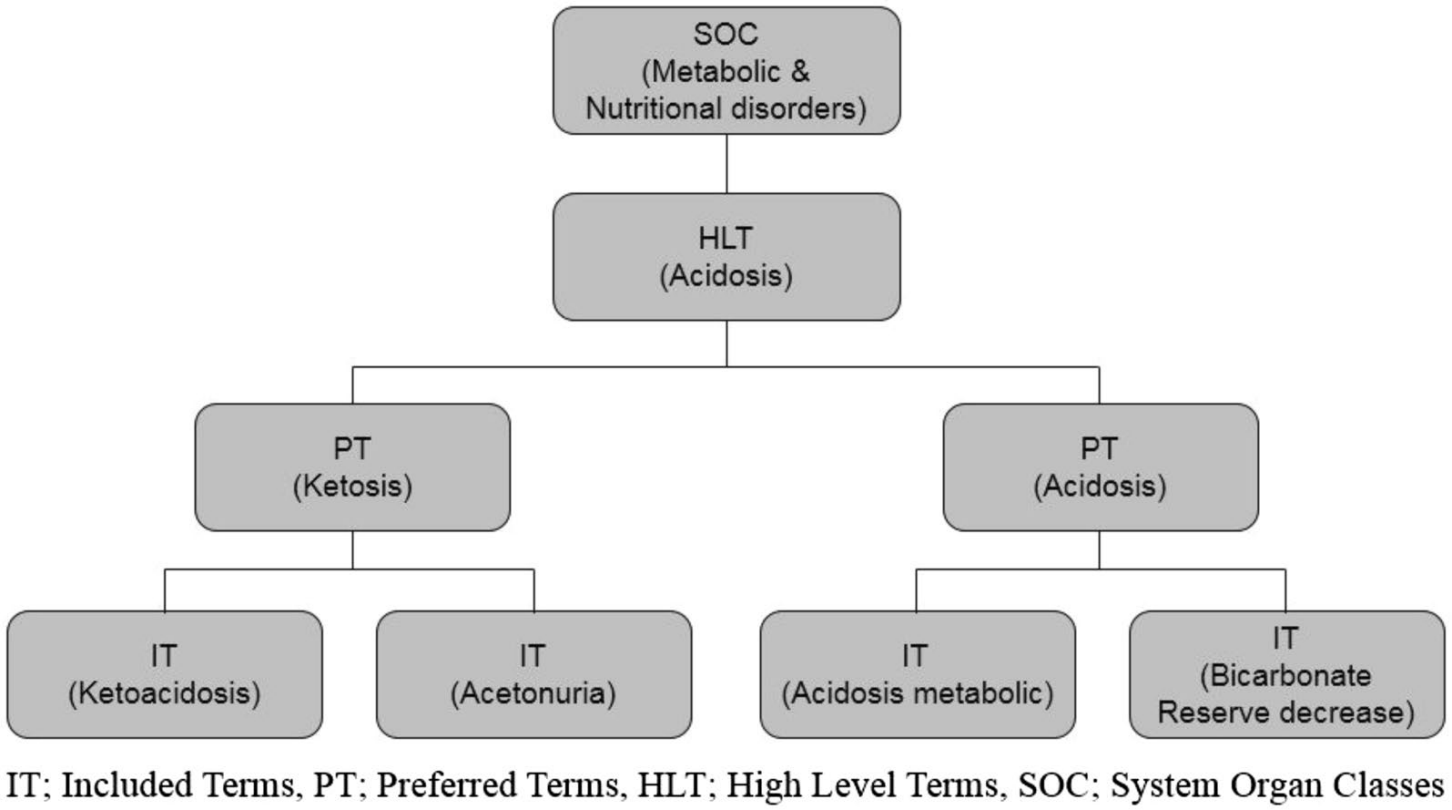
WHO-ART structure.

In pharmacovigilance data, AE information uses adverse reaction terms, which have a hierarchical structure. For example, as shown in Fig. 1, the WHO Adverse Reaction Terminology (WHO-ART) developed for the WHO drug monitoring program has a four-level hierarchical structure. (https://www.who-umc.org) Owing to this type of structure, it is difficult to determine the level of AE definition that should be used during data mining. To solve the problem, tree-based scan statistics, which find signals at each level of AEs in the form of a hierarchical tree, have been proposed by Kulldorff et al.^26^ and have been recently used by some researchers to detect AE signals^27–29^. The tree-based scan statistic is distinct from most disproportionality methods; it is based on scan statistical theory and uses a hierarchical diagnosis tree to simultaneously assess risk at any level of granularity, adjusting for a multiple testing problem in several overlapping evaluated groups^7,26,30^.

Most of these AE databases have large numbers of zero-count cells. For example, AERS data from 2006 to 2011 show that the percentage of zero-count cells by the drug ranges from 50 to 99.99%^31^. However, based on KAERS data from 2012 to 2016, the percentage of zero-count cells by the drug ranges from 75 to 100%. Zero-count cells may contain not only zero-valued observations from the Poisson distribution, but also true zeros, which represent theoretically impossible observations or possible but unreported observations. Data with a large number of zeros cannot be assumed to have a Poisson distribution as some zeros are true zeros. The distribution of such data is typically more dispersed than the Poisson distribution, resulting in equality between the variance and the mean of the distribution. To solve this problem, the zero-inflated Poisson (ZIP) model proposed by Lambert^32^ can be used. Huang et al.^31,33^ proposed a zero-inflated Poisson model based likelihood ratio test (ZIP-LRT) method as an extended version of LRT, a frequentist data mining method. Further, Hu et al.^24^ developed the zero-inflated Poisson simplified Bayes method and the zero-inflated Poisson Dirichlet process method, which are Bayesian data mining methods.

The existing tree-based scan statistic assumes all zero values are zero-valued observations from the Poisson distribution. As a result, true zeros are not considered in the modeling, which can lead to bias in the inferences. Therefore, in this study, we proposed a new tree-based scan statistic using the ZIP model for data with excess zeros in a hierarchical structure.

In section “A tree-based scan statistic”, we introduce the existing tree-based scan statistic. In section “A tree-based scan statistic for zero inflated count data”, we propose a tree-based scan statistic for zero-inflated count data. In section “Simulation study”, a simulation study to evaluate the performance of the proposed method is presented. In section “Real data”, the two methods are compared through a real data example. Finally, in section “Conclusion and discussion”, we summarize the results and conclude with our recommendations.

### Hierarchical diagnosis tree

The tree-based scan statistic uses hierarchical classification systems to represent clinical concepts, such as drugs, procedures, or diagnoses^30^. To code adverse drug reactions in postmarket drug surveillance, medical terminologies, such as Medical Dictionary for Regulatory Activities (MedDRA) and WHO-ART, are used. In the KEARS data, WHO-ART is used to code the AEs.

WHO-ART is the terminology for coding clinical information related to pharmacotherapy and is commonly used for coding the AEs. When new drugs and new symptoms create new terms that incorporate them, the structure of the terms is updated to include the newly integrated terms while retaining their previous relationships and the existing structure of terms. WHO-ART has a four-level hierarchical structure, which consists of System Organ Class (SOC), High Level Terms (HLT), Preferred Terms (PT), and Included Terms (IT). The highest level, the SOC, corresponds to body systems and organs, which contain grouping terms. The HLT is used to group related or similar PTs, but all PTs are not grouped into the HLT. The PTs are principal terms used to describe AEs and the ITs are synonyms of the PTs, which help in the search for the PTs. An example of the WHO-ART is shown in Table 1.

**Table 1:** Example of WHO-ART.

SOC	PT	IT	Diagnosis	HLT
0800			Metabolic and nutritional disorders	
0800	0363		Acidosis	0363
0800	0363	003	Bicarbonate reserve decreased	0363
0800	0363	004	PH reduced	0363
0800	0363	005	Acidosis Metabolic	0363
0800	0363	006	Blood bicarbonate decreased	0363
0800	0363	007	Blood PH decreased	0363
0800	0363	008	Acidosis hyperchloraemic	0363
0800	0364		Acidosis lactic	0363
0800	0364	003	Lactate blood increase	0363
0800	0393		Ketosis	0363
0800	0393	003	Ketoacidosis	0363
0800	0393	004	Acetonuria	0363
0800	0393	005	Acetone breath	0363
0800	0393	006	Acetonaemia	0363
0800	0393	007	Diabetic ketoacidosis	0363
0800	1465		Acidosis respiratory	0363
0800	1465	002	Blood carbon dioxide increased	0363

## A tree-based scan statistic

### Review of a tree-based scan statistic

The tree-based scan statistic is a statistical data mining method that has been used for signal detection in a hierarchically structured data, such as a classification system for coding AEs. This statistic searches signals at any level of AE definitions, called leaves. Each leaf contains information on the total number of patients with a specific AE and the number of patients with a specific AE from a certain drug. Mutually-related leaves are grouped into a higher level, called a node. Of note, a cut defines a branch of the tree where a node or a leaf may have more events than expected.

The tree-based scan statistic method considers all possible cuts. For each cut, the total number of AEs from all drugs and a certain drug are respectively calculated for the leaves within that cut. The test statistic is generated by a likelihood function in which risk is estimated separately for the leaves defined by the cut and those outside of the cut^26,34,35^.

Let ${c}_{i}$ be the observed number of patients with *i*th AE potentially caused by a certain drug in leaf $i$ and ${n}_{i}$ be the total observed number of patients with $i$ th AE in leaf $i$. For a rare disease, with covariates ignored, ${c}_{i}$ is approximately Poisson distributed with mean ${n}_{i}{\lambda }_{i}$, where ${\lambda }_{i}$ is the probability that $i$ th AE is caused by a certain drug. For all leaves on the tree, let $C={\sum }_{i=1}^{I}{c}_{i}$ and $N={\sum }_{i=1}^{I}{n}_{i}$ where *I* is the number of all leaves in the tree. For each cut *G*, a leaf or a group of related leaves, let ${c}_{G}={\sum }_{i\in G}{c}_{i}$ and ${n}_{G}={\sum }_{i\in G}{n}_{i}$. *R* is the rest of the leaves except those included in *G*. The following null hypothesis ${H}_{0}: {\lambda }_{G}={\lambda }_{0}$ and the alternative hypothesis ${H}_{a}:{\lambda }_{G}>{\lambda }_{R}$ are considered. The null hypothesis suggests that the probability that AEs in a cut *G* due to a certain drug are not lower or higher than that of all AEs. The alternative hypothesis is that at least one cut is defined by a set *G* such that ${\lambda }_{G}>{\lambda }_{R}$, where *R* is a group of the remaining leaves.

Of note, the analysis is only concerned with *C*, as the total number of AEs represented by the tree is not of interest. In fact, only the relative distribution between the different AEs is relevant. The likelihood can then be expressed as $L\left(\lambda ,{\varvec{c}}\right)=\prod_{i}{\left(\frac{{n}_{i}{\lambda }_{i}}{{\sum }_{i}{n}_{i}{\lambda }_{i}}\right)}^{{c}_{i}}$ using a multinomial distribution. As a maximum likelihood estimator (MLE) of ${\lambda }_{G}/{\lambda }_{R}$ is $\frac{{c}_{G}/{n}_{G}}{(C-{c}_{G})/(N-{n}_{G})}$ given *G,* a likelihood ratio test statistic is $T= \frac{\underset{G, {\lambda }_{G}>{\lambda }_{R}}{\mathrm{max}}L\left(\lambda , {\varvec{c}}\right)}{\underset{ {\lambda }_{G}={\lambda }_{R}}{\mathrm{max}}L\left(\lambda , {\varvec{c}}\right)}={\left(\frac{N}{C}\right)}^{C}\underset{G}{\mathrm{max}}{\left(\frac{{c}_{G}}{{n}_{G}}\right)}^{{c}_{G}}{\left(\frac{C-{c}_{G}}{N-{n}_{G}}\right)}^{C-{c}_{G}}$ when $\frac{{c}_{G}}{{n}_{G}}>\frac{C-{c}_{G}}{N-{n}_{G}}$; otherwise, the statistic is 1. The log-likelihood ratio-based test statistic is given by

$$\mathrm{log}T=\underset{G}{\mathrm{max}}\left\{{c}_{G}\mathit{log}\left(\frac{{c}_{G}}{{n}_{G}}\right)+\left(C-{c}_{G}\right)\mathit{log}\left(\frac{C-{c}_{G}}{N-{n}_{G}}\right)\right\}\times I\left(\frac{{c}_{G}}{{n}_{G}}>\frac{C-{c}_{G}}{N-{n}_{G}}\right),$$

where *I*() is the indicator function^26^.

### Hypothesis testing

To calculate the test statistic T, the likelihood of each possible cut was determined. The cut, which is maximizing the likelihood ratio value, is defined as the most likely cut; the likelihood ratio value is defined as the test statistic T. As the null distribution of the test statistic is unknown, it is produced using the Monte Carlo simulation^36^. Given the total number of patients with AEs from a certain drug, a large number of random data sets was created under the null hypothesis, and the test statistics for each random data set and the real data were calculated. The obtained test statistics for random datasets were compared to the test statistic for the real data. The *P*-value was calculated using the equation: rank/(1 + B), where rank is the relative position of the test statistic for the real data among the test statistics for the random data sets and B is the number of Monte Carlo replications.

## A tree-based scan statistic for zero-inflated count data

In the presence of excess zero, the Poisson model tends to underestimate the observed dispersion. In this case, the ZIP model can be employed as one of the approaches to resolve the problem as this model is more flexible than the Poisson model. If the number of *i*th AE with a certain drug ${C}_{i}$ follows the ZIP model, with the probability *p* of a true zero and the average number of events ${n}_{i}{\lambda }_{i}$, ${C}_{i} \sim \mathrm{ZIP}(p, {n}_{i}{\lambda }_{i})$, the mean and variance can be expressed as $E({C}_{i}\left|p, {n}_{i}{\lambda }_{i}\right)=(1-p){n}_{i}{\lambda }_{i}$ and $V({C}_{i}\left|p, {n}_{i}{\lambda }_{i}\right)=(1-p){n}_{i}{\lambda }_{i}(1+p{n}_{i}{\lambda }_{i})$. It can also be expressed as $V({C}_{i}\left|p, {n}_{i}{\lambda }_{i}\right)=E({C}_{i}\left|p, {n}_{i}{\lambda }_{i}\right)(1+p{n}_{i}{\lambda }_{i})$; thus, $V({C}_{i}\left|p, {n}_{i}{\lambda }_{i}\right)>E({C}_{i}\left|p, {n}_{i}{\lambda }_{i}\right)$ when *p* > 0.

As the ZIP model has an additional parameter relative to the tree-based scan statistic, its mean is smaller than that of the Poisson model. Thus, the ZIP model correctly calculates a reduced number of *i*th AEs with a certain drug due to the presence of true zeros.

Given the parameters $p$ and ${n}_{i}{\lambda }_{i}$, the probability of ${C}_{i}={c}_{i}$ is described as follows:

$$P({C}_{i}={c}_{i}\left|p, {n}_{i}{\lambda }_{i}\right)=\left\{\begin{array}{c}\begin{array}{cc}p+\left(1-p\right){e}^{-{n}_{i}{\lambda }_{i}}& , {c}_{i}=0\end{array} \\ \begin{array}{cc}\left(1-p\right)\frac{{e}^{{-n}_{i}{\lambda }_{i}}{\left({n}_{i}{\lambda }_{i}\right)}^{{c}_{i}}}{{c}_{i}!}& , {c}_{i}>0.\end{array}\end{array}\right.$$

For the tree-based ZIP scan statistic, the hypotheses of interest are the same as those in section “Review of a tree-based scan statistic”. The zeros are assumed to be known, whether or not they are true zeros, as it is difficult to find a closed form of MLE when the nature of each zero is unknown. As tree-based scan statistics are based on scan statistic theory, the methodology of Cançado et al.^37^, who proposed a spatial scan statistical method for zero-inflated Poisson processes, was employed.

We consider a vector $\delta =({\delta }_{1},\dots , {\delta }_{I})$ where ${\delta }_{i}=1$ for a true zero in leaf $i$ and ${\delta }_{i}=0$ for an observational zero in leaf $i$. ${\delta }_{i}$ s are Bernoulli random variables with the probability *p* of a true zero. Given a set of observations $\delta =({\delta }_{1},\dots ,{\delta }_{I})$ that are bivariate data such that $\left({C}_{i}, {\delta }_{i}\right)$, $i=1,\dots ,I$, the likelihood function for set *G* can be expressed as

$$L\left(p, {\lambda }_{R}, {\lambda }_{G}\right)=\left[\prod_{i\in G}{p}^{{d}_{i}}{\left[\left(1-p\right)\frac{{e}^{{-n}_{i}{\lambda }_{i}}{\left({n}_{i}{\lambda }_{G}\right)}^{{c}_{i}}}{{c}_{i}!}\right]}^{(1-{d}_{i})}\right]\left[\prod_{i\notin G}{p}^{{d}_{i}}{\left[\left(1-p\right)\frac{{e}^{{-n}_{i}{\lambda }_{i}}{\left({n}_{i}{\lambda }_{R}\right)}^{{c}_{i}}}{{c}_{i}!}\right]}^{(1-{d}_{i})}\right].$$

When ${\delta }_{i}$ s are known, the MLEs under the null hypothesis are ${\widehat{\lambda }}_{0}=\frac{{\sum }_{i=i}^{I}{c}_{i}\left(1-{d}_{i}\right)}{{\sum }_{i=i}^{I}{n}_{i}\left(1-{d}_{i}\right)}\mathrm{ and }{\widehat{p}}_{0}=\frac{{\sum }_{i=i}^{I}{d}_{i}}{I}$. However, under the alternative hypothesis, the MLEs are ${\widehat{\lambda }}_{G}=\frac{{\sum }_{i\in G}{c}_{i}\left(1-{d}_{i}\right)}{{\sum }_{i\in G}{n}_{i}\left(1-{d}_{i}\right)}, {\widehat{\lambda }}_{R}=\frac{{\sum }_{i\notin G}{c}_{i}\left(1-{d}_{i}\right)}{{\sum }_{i\notin G}{n}_{i}\left(1-{d}_{i}\right)},\mathrm{ and }\widehat{p}=\frac{{\sum }_{i=i}^{I}{d}_{i}}{I}$.

When ${\delta }_{i}$ s are unknown, an expectation–maximization (EM) algorithm is used to find the MLEs of ${\lambda }_{0},{\lambda }_{G}, {\lambda }_{R}, {p}_{0}$ and $p$. In the expectation step (E-step), the expected value of ${\delta }_{i}$, given ${C}_{i}$, is calculated using the following formula:

$${\widehat{\delta }}_{i}^{(m)}=\frac{{\widehat{p}}^{\left(m\right)}}{{\widehat{p}}^{\left(m\right)}+\left(1-{\widehat{p}}^{\left(m\right)}\right){e}^{-{n}_{i}{\widehat{\lambda }}_{0}^{m}}}I\left({c}_{i}=0\right), i=1, \dots , I.$$

Under ${H}_{a}$, ${\widehat{\lambda }}_{0}$ is considered ${\widehat{\lambda }}_{G}$ and ${\widehat{\lambda }}_{R}$ in each cut *G* and the remaining leaves R, respectively.

In the maximization step (M-step), the MLEs of ${\lambda }_{0},{\lambda }_{G}, {\lambda }_{R}, {p}_{0}$, and $p$ are updated via the equations with ${d}_{i}$ replaced by ${{\widehat{\delta }}_{i}}^{\left(m\right)}$ when ${\delta }_{i}$ s are known. Until the maximum likelihood estimates for each possible cut *G* converge, the above E- and M-steps are performed repeatedly. To perform a faster calculation, we used the ‘zeroinfl’ function in the R package “pscl”^38^. For the possible candidate cuts, this process should be conducted and the most likely cut should be determined.

The likelihood ratio for cut *G* can be expressed as

$$L{R}_{G}=\frac{{\left[\frac{\sum_{i\in G}{c}_{i}\left(1-{d}_{i}\right)}{\sum_{i\in G}{n}_{i}\left(1-{d}_{i}\right)}\right]}^{\sum_{i\in G}{c}_{i}\left(1-{d}_{i}\right)}{\left[\frac{\sum_{j\notin G}{c}_{j}\left(1-{d}_{j}\right)}{\sum_{j\notin G}{n}_{j}\left(1-{d}_{j}\right)}\right]}^{\sum_{j\notin G}{c}_{j}\left(1-{d}_{j}\right)}}{{\left[\frac{\sum_{i=1}^{I}{c}_{i}\left(1-{d}_{i}\right)}{\sum_{i=1}^{I}{n}_{i}\left(1-{d}_{i}\right)}\right]}^{\sum_{i=1}^{I}{c}_{i}\left(1-{d}_{i}\right)}}\times I\left(\frac{\sum_{i\in G}{c}_{i}\left(1-{d}_{i}\right)}{\sum_{i\in G}{n}_{i}\left(1-{d}_{i}\right)}>\frac{\sum_{j\notin G}{c}_{j}\left(1-{d}_{j}\right)}{\sum_{j\notin G}{n}_{j}\left(1-{d}_{j}\right)}\right).$$

Thereafter, the maximum likelihood ratio is defined as the test statistic, $T=\underset{G}{\mathrm{max}}L{R}_{G}.$

As it is impossible to know the null distribution of the likelihood ratio test statistic *T*, Monte Carlo hypothesis testing was conducted to assess statistical significance^37^.

## Simulation study

### Data generating process and performance assessment measures

We conducted a simulation study to assess the performance of the proposed tree-based scan statistic for zero-inflated count data (TreeScan-ZIP) and the existing tree-based scan statistic (TreeScan-Poisson). For the simulation study, datasets with the hierarchical structure where AEs can be expressed in terms of WHO-ART SOCs and PTs were generated. Only 105 of the 1292 AEs in the PT terms were considered to reduce computation time. Different artificial true signals and true zeros were generated using a tree with 105 leaves and 9 nodes. The total numbers of patients with each AE varied from 10 to 4670. The total number of patients in all leaves of the tree was 19,920 and the total number of patients with AEs from a certain drug was 640.

First, true zeros $({\delta }_{i}=1)$ were randomly allocated using the Bernoulli distribution with the probability *p*, where *p* is the percentage of the true zero leaves. Thereafter, for each iteration, the total number of patients with AEs from a certain drug, that is $C={\sum }_{i=1}^{I}{c}_{i}$, was randomly assigned to the leaves on the tree as multinomial, with probabilities proportional to the relative risk. The relative risk of *i*th leaf was computed as $\frac{{c}_{i}/{n}_{i}}{C/N}, i=1,\dots ,I.$ For true zero leaves, $\left({\delta }_{i}=1\right), {c}_{i}=0$. If the *i*th leaf was not a true zero, the dataset was generated using a multinomial distribution. Under *H*_*0*_, the vector ${\varvec{C}}=({c}_{1},\dots , {c}_{I})$ follows a multinomial distribution with parameters ${\varvec{C}}$ and ${\varvec{p}}$, where ${\varvec{p}}=\left(\frac{{n}_{1}}{N}, \dots ,\frac{{n}_{I}}{N}\right)$. Under ${H}_{a}$, ${\varvec{p}}=\left(\frac{r{r}_{1}\frac{{n}_{1}}{N}}{{\sum }_{i=1}^{I}r{r}_{i}\frac{{n}_{i}}{N}}, \dots , \frac{r{r}_{I}\frac{{n}_{I}}{N}}{{\sum }_{i=1}^{I}r{r}_{i}\frac{{n}_{i}}{N}}\right)$, where $r{r}_{1},\dots ,r{r}_{I}$ are the relative risks of all types of AEs. The relative risk of the randomly selected true signal leaves ranged from 3, 4, and 2 to 6; however, for the other leaves, except the true zero leaves, the relative risk was equal to 1.

Based on the total number of cases, *C* = 640, we considered 0, 10, 30, 50, and 70 for the number of true zero leaves, and 1%, 3%, 5%, and 10% for the true signal leaves with the relative risk (RR). All possible combinations were simulated.

To evaluate the performance of the two methods, we computed type I error, power, sensitivity, and positive predicted value (PPV). First, the critical value T* was obtained from 10,000 random datasets under *H*_*0*_ by the Monte Carlo replications for each scenario according to the number of true zeros (0, 10, 30, 50, 70). Thereafter, *B* random datasets were generated under ${H}_{0}$ and ${H}_{a}$ to calculate type I error, power, sensitivity, and PPV. For each of the *B* random datasets, test statistic ${T}_{k}, k=1,\dots ,B,$ was calculated using both methods.

Thereafter, type I error and power were estimated using

$$\mathrm{Type\, I \,error}=\frac{{\sum }_{k=1}^{B}I({T}_{k}>{T}^{*}|{H}_{0})}{B}$$

$$\mathrm{Power}=\frac{{\sum }_{k=1}^{B}I({T}_{k}>{T}^{*}|{H}_{a})}{B}.$$

Sensitivity and PPV for each random datasets are expressed as

$$\mathrm{Sensitivity}=\frac{\#\,\mathrm{ of }(\mathrm{detected \,signal}\cap \mathrm{true\, signal})}{\#\,\mathrm{ of }(\mathrm{true\, signal})},$$

$$\mathrm{PPV}=\frac{\#\,\mathrm{ of }(\mathrm{detected\, signal}\cap \mathrm{true\, signal})}{\#\,\mathrm{ of }(\mathrm{detected\, signal})}.$$

Overall sensitivity and PPV were calculated as the average of sensitivity and PPV over ${B}^{^{\prime}}$ random datasets, where ${B}^{^{\prime}}={\sum }_{k=1}^{B}I({T}_{k}>{T}^{*})$.

### Results

The results obtained using the simulated data are presented in Table 2. The type I errors for the TreeScan-Poisson and TreeScan-ZIP methods were close to 0.05, except when the data had a Poisson distribution. The type I error of the TreeScan-Poisson method was above the nominal significance level of 0.05, while the type I error of the TreeScan-ZIP method tended to be less than 0.05.

**Table 2:** Type I error, power, sensitivity and positive predictive value obtained by the two methods according to the number of true signals and relative risk.

True zero	True signal	RR	TreeScan-Poisson			TreeScan-ZIP		
			Power*	Sensitivity	PPV	Power*	Sensitivity	PPV
0	0		0.043			0.043		
	2	3	0.061	0.144	0.275	0.061	0.144	0.275
	2	4	0.086	0.260	0.490	0.086	0.261	0.491
	2	(3.8, 6)	0.125	0.343	0.654	0.126	0.339	0.648
	6	3	0.833	0.176	0.976	0.833	0.176	0.976
	6	4	0.988	0.192	0.984	0.989	0.192	0.983
	6	(3.8, 6)	1.000	0.209	0.987	1.000	0.209	0.986
	7	3	1.000	0.379	0.995	1.000	0.380	0.995
	7	4	1.000	0.429	0.997	1.000	0.429	0.997
	7	(3.8, 6)	1.000	0.451	0.997	1.000	0.451	0.997
	13	3	1.000	0.234	0.996	1.000	0.235	0.996
	13	4	1.000	0.297	0.998	1.000	0.299	0.998
	13	(3.8, 6)	1.000	0.386	0.999	1.000	0.389	0.999
10	0		0.052			0.046		
	2	3	0.047	0.000	0.000	0.070	0.173	0.338
	2	4	0.042	0.000	0.000	0.104	0.304	0.581
	2	(3.8, 6)	0.040	0.000	0.000	0.159	0.388	0.732
	6	3	0.002	0.069	0.417	0.952	0.192	0.983
	6	4	0.085	0.166	0.993	1.000	0.241	0.988
	5	(3.8, 6)	0.901	0.200	1.000	1.000	0.388	0.992
	7	3	1.000	0.286	1.000	1.000	0.398	0.998
	7	4	1.000	0.286	1.000	1.000	0.434	0.999
	8	(3.8, 6)	1.000	0.276	1.000	1.000	0.389	0.999
	13	3	0.996	0.153	1.000	1.000	0.250	0.998
	13	4	1.000	0.154	1.000	1.000	0.318	0.999
	13	(3.8, 6)	1.000	0.164	1.000	1.000	0.404	1.000
30	0		0.051			0.049		
	2	3	0.044	0.000	0.000	0.071	0.205	0.390
	2	4	0.037	0.000	0.000	0.115	0.338	0.629
	2	(3.8, 6)	0.035	0.000	0.000	0.180	0.404	0.742
	6	3	0.002	0.000	0.000	0.982	0.242	0.986
	6	4	0.000	0.000	0.000	1.000	0.339	0.991
	5	(3.8, 6)	0.174	0.200	1.000	1.000	0.452	0.993
	8	3	0.830	0.232	1.000	1.000	0.359	0.998
	8	4	1.000	0.250	1.000	1.000	0.388	0.999
	8	(3.8, 6)	1.000	0.250	1.000	1.000	0.421	0.999
	14	3	0.733	0.128	1.000	1.000	0.258	0.998
	14	4	1.000	0.143	1.000	1.000	0.326	0.999
	14	(3.8, 6)	1.000	0.143	1.000	1.000	0.407	1.000
50	0		0.052			0.051		
	2	3	0.040	0.000	0.000	0.103	0.298	0.543
	2	4	0.033	0.000	0.000	0.184	0.423	0.761
	2	(3.8, 6)	0.023	0.000	0.000	0.313	0.519	0.875
	6	3	0.001	0.000	0.000	0.998	0.349	0.990
	6	4	0.000	0.167	1.000	1.000	0.397	0.993
	6	(3.8, 6)	0.258	0.167	1.000	1.000	0.404	0.995
	8	3	0.969	0.247	1.000	1.000	0.409	0.998
	8	4	1.000	0.250	1.000	1.000	0.464	0.999
	8	(3.8, 6)	1.000	0.250	1.000	1.000	0.529	1.000
	14	3	0.908	0.138	1.000	1.000	0.287	1.000
	14	4	1.000	0.143	1.000	1.000	0.362	1.000
	14	(3.8, 6)	1.000	0.143	1.000	1.000	0.466	1.000
70	0		0.050			0.049		
	2	3	0.040	0.000	0.000	0.226	0.499	0.826
	2	4	0.035	0.000	0.000	0.462	0.586	0.926
	2	(3.8, 6)	0.033	0.000	0.000	0.735	0.664	0.967
	6	3	0.000	–	–	1.000	0.417	0.996
	6	4	0.013	0.167	1.000	1.000	0.499	0.997
	6	(3.8, 6)	0.969	0.167	1.000	1.000	0.527	0.999
	7	3	1.000	0.286	1.000	1.000	0.429	0.999
	7	4	1.000	0.286	1.000	1.000	0.477	0.999
	7	(3.8, 6)	1.000	0.286	1.000	1.000	0.586	0.999
	13	3	1.000	0.154	1.000	1.000	0.249	0.999
	13	4	1.000	0.154	1.000	1.000	0.294	0.999
	13	(3.8, 6)	1.000	0.143	1.000	1.000	0.466	1.000

* Type I error when the number of true signals is 0.

When the data did not include true zeros (i.e., the data were generated from the Poisson distribution), the TreeScan-Poisson and TreeScan-ZIP methods produced similar power, sensitivity, and PPV estimates.

The TreeScan-ZIP method was identified to produce higher power and sensitivity estimates than the TreeScan-Poisson method when the number of true zeros was greater than or equal to 10. In the presence of zero inflation, when the number of true signals was greater than or equal to 5 and the RR was high, the PPV of the TreeScan-Poisson method was 1.0. The TreeScan-Poisson method could detect highly significant cuts, resulting in a small number of detected signals, which indicated high PPV and low sensitivity.

The TreeScan-ZIP method performed better than the TreeScan-Poisson in every dataset with true zero. The estimated power was almost 1.0 and the PPV was greater than 0.98 when the number of true zeros was greater than or equal to 10 and the number of true signals was greater than or equal to 5. The TreeScan-ZIP method was more sensitive than the TreeScan-Poisson method. The sensitivity and PPV of the TreeScan-ZIP method became higher with higher RR. When two true signals existed, both methods had a relatively low power; however, the power of the TreeScan-ZIP method increased as the number of true zeros and RR increased.

The simulation study showed that in the presence of zero inflation, the TreeScan-ZIP method performed better than the TreeScan-Poisson method.

## Real data

### Korea adverse event reporting system data

KAERS is a spontaneous AE reporting system maintained by the Korea Institute of Drug Safety and Risk Management (https://www.drugsafe.or.kr). Consumers, Healthcare Professionals, Regional Pharmacovigilance Centers (RPVCs), and pharmaceutical companies can report suspected drug information and AE information using the KAERS. RPVCs evaluate causality between the suspected drug and AE and report them to KIDS. The information is then stored in the KAERS as an individual case safety report (ICSR), which contains information on suspected drug, AE, causal relationship, and demographic. The ICSRs are periodically summited to the WHO-UMC. Further, safety information obtained from KAERS data and signal analysis is periodically reported to the Ministry of Food and Drug Safety.

For the real data analysis, data cleansing was performed. Because a certain drug and AE information can be reported multiple times depending on the dose and time of administration, if the same drug and AE were reported twice or more, only the first report was used. In the causality, only drug–AE pairs that received ratings of possible or above were included in this study. There are 6 levels of causality: certain, probable, possible, unlikely, conditional, and unassessable^39,40^. In KAERS database, AEs are coded by the WHO-ART. As more than half of the reports included information down to the PT level, and HLT may not exist, this study used two levels of hierarchy, SOC and PT, with the exception of the HLT and IT level.

Data obtained between 2012 and 2016 from KAERS were used. During this period, 716,584 people reported experiencing AEs. There were 1.8 million drug reports on 1981 types of drugs and 1.1 million AE reports on 4078 types of AEs. Further, a total of 2.4 million unique drug-AE pairs were found. When removing pairs that had beneath the ‘possible’ threshold, the final dataset analyzed in this study included 1,077,060 drug-AE pairs representing 1292 types of AEs in PTs. Further, 1981 types of drugs were identified in 557,390 reports.

### Paclitaxel and docetaxel

The two proposed methods were applied to detect the AE signals to the drug–AE pairs data from KAERS. Paclitaxel and docetaxel, which have the highest sales among all anticancer drugs in the world, were selected^41^. Of note, these are representatives of the new class of taxane drugs, which have emerged as a fundamental treatment for breast cancer. Paclitaxel and docetaxel have similar main structures and mechanisms of action^42^. Paclitaxel is used to treat a number of cancer types, including Kaposi sarcoma, breast cancer, ovarian cancer, lung cancer, cervical cancer, and pancreatic cancer (https://www.ashp.org/). Docetaxel is also used as to treat several cancer types, including breast cancer, non-small cell lung cancer, prostate cancer, head and neck cancer, and stomach cancer (https://www.cancer.gov/). The most frequently reported AEs related to taxene from MICROMEDEX® include cardiovascular effects, dermatologic effects, endocrine/metabolic effects gastrointestinal effects, hematologic effects, hepatic effects, immunologic effects, musculoskeletal effects, neurologic effects, ophthalmic effects, otic effects, renal effects, respiratory effects, and others (https://www.who.int/).

### Results

#### Paclitaxel

Nine signals were identified by the TreeScan-Poisson method and 30 signals were detected by the TreeScan-ZIP method (Table 3). The nine signals detected by the TreeScan-Poisson method were also detected by the TreeScan-ZIP method. The AEs corresponding to the signals found by both methods were related to the following SOCs: central & peripheral nervous system disorders (0410), respiratory system disorders (1100), white cell and reticuloendothelial system disorders (1220), and body as a whole—general disorders (1810). Further, their PTs were paresthesia (0410.0137), neuropathy peripheral (0410.1313), dyspnea (1100. 0514), granulocytopenia (1220.0572), leucopenia (1220.0908), chest pain (1810.0718), and temperature change sensations (1810.1705). The TreeScan-ZIP method detected signals related to 10 SOC terms. The nine signals detected by the two methods were included in the known AEs. However, some signals detected by TreeScan-ZIP alone were included in the known AEs.

**Table 3:** Results of signal detection of adverse events of paclitaxel by the two methods.

SOC	PT	Diagnosis	Marginal total	Obs	TreeScan-Poisson			TreeScan-ZIP		
					Exp	O/E	p-value	Exp	O/E	p-value
0100		Skin and appendages disorders	352,949	1020	1176.5	0.9	1.000	868.3	1.2	1.000
	0002	ALOPECIA	13,193	94	44.0	2.1	0.929	32.5	2.9	0.001
	0043	SWEATING INCREASED	13,433	100	44.8	2.2	0.890	33.0	3.0	0.001
	0828	HYPOTRICHOSIS	234	13	0.8	16.7	0.897	0.6	22.6	0.001
0200		Musculo-skeletal system disorders	50,667	305	168.9	1.8	0.639	124.6	2.4	0.001
	0073	MYALGIA	24,679	244	82.3	3.0	0.119	60.7	4.0	0.001
0410		Central & peripheral nervous system disorders	233,135	781	777.1	1.0	1.000	573.5	1.4	1.000
	0117	HYPOAESTHESIA	2349	32	7.8	4.1	0.934	5.8	5.5	0.001
	0130	NEUROPATHY	4198	81	14.0	5.8	0.318	10.3	7.8	0.001
	0137	PARAESTHESIA	12,165	213	40.5	5.3	0.015	29.9	7.1	0.001
	1313	NEUROPATHY PERIPHERAL	5634	150	18.8	8.0	0.015	13.9	10.8	0.001
	2082	POLYNEUROPATHY	183	7	0.6	11.5	0.998	0.5	15.5	0.001
0800		Metabolic and nutritional disorders	68,669	92	228.9	0.4	1.000	168.9	0.5	1.000
	0368	CACHEXIA	3184	49	10.6	4.6	0.754	7.8	6.3	0.001
1030		Heart rate and rhythm disorders	22,346	206	74.5	2.8	0.270	55.0	3.7	0.001
	0221	PALPITATION	11,778	99	39.3	2.5	0.810	29.0	3.4	0.001
	0224	TACHYCARDIA	4569	94	15.2	6.2	0.177	11.2	8.4	0.001
1040		Vascular (extracardiac) disorders	13,671	100	45.6	2.2	0.897	33.6	3.0	0.001
	0207	FLUSHING	5334	91	17.8	5.1	0.312	13.1	6.9	0.001
1100		Respiratory system disorders	137,936	588	459.8	1.3	0.961	339.3	1.7	0.001
	0514	DYSPNOEA	36,735	410	122.4	3.3	0.010	90.4	4.5	0.001
	0537	RESPIRATORY INSUFFICIENCY	1292	18	4.3	4.2	0.996	3.2	5.7	0.001
1220		White cell and RES disorders	92,531	1085	308.4	3.5	0.001	227.6	4.8	0.001
	0570	AGRANULOCYTOSIS	8089	89	27.0	3.3	0.656	19.9	4.5	0.001
	0572	GRANULOCYTOPENIA	58,735	674	195.8	3.4	0.001	144.5	4.7	0.001
	0908	LEUCOPENIA	20,456	314	68.2	4.6	0.008	50.3	6.2	0.001
1700		Neoplasms	6336	25	21.1	1.2	1.000	15.6	1.6	0.996
	1345	NEOPLASM MALIGNANT	591	13	2.0	6.6	0.989	1.5	8.9	0.001
1810		Body as a whole—general disorders	220,690	1295	735.6	1.8	0.011	542.9	2.4	0.001
	0712	ALLERGIC REACTION	1394	18	4.6	3.9	0.998	3.4	5.2	0.001
	0718	CHEST PAIN	25,856	479	86.2	5.6	0.001	63.6	7.5	0.001
	0730	PAIN	7221	54	24.1	2.2	0.987	17.8	3.0	0.001
	1705	TEMPERATURE CHANGED SENSATION	9204	245	30.7	8.0	0.003	22.6	10.8	0.001
	2237	ANAPHYLACTIC REACTION	3680	44	12.3	3.6	0.895	9.1	4.9	0.001

#### Docetaxel

The TreeScan-Poisson and the TreeScan-ZIP methods identified 9 and 56 signals, respectively (Table 4). All signals detected by the TreeScan-Poisson method were also detected by the TreeScan-ZIP method. The AEs corresponding to the signals found by both methods were related to the following SOCs: skin and appendages disorders (0100), musculo-skeletal system disorders (0200), central & peripheral nervous system disorders (0410), red blood cell disorders (1210), white cell and reticulo-endothelial system (RES) disorders (1220). Their PTs were alopecia (0100.0002), nail disorder (0100.0020), myalgia (0200.0073), sensory disturbance (0410.0148), anemia (1210.0544), and granulocytopenia (1220.0572). The TreeScan-ZIP method detected signals related to 18 SOC terms. All signals detected by the two methods were included in the known AEs. A few signals that were not detected by TreeScan, but were detected by TreeScan-ZIP, were included in known AEs, such as vision disorders, gastro-intestinal system disorders, liver and biliary system disorders, urinary system disorders, etc.

**Table 4:** Results of signal detection of adverse events of docetaxel by the two methods.

SOC	PT	Diagnosis	Marginal total	Obs	TreeScan-Poisson			TreeScan-ZIP		
					Exp	O/E	*p*-value	Exp	O/E	*p*-value
0100		Skin and appendages disorders	352,949	4420	5126.4	0.9	1.000	3467.1	1.3	1.000
	0002	ALOPECIA	13,193	2212	191.6	11.5	0.001	129.6	17.1	0.001
	0008	DERMATITIS EXFOLIATIVE	161	11	2.3	4.7	1.000	1.6	7.0	0.024
	0020	NAIL DISORDER	3248	760	47.2	16.1	0.005	31.9	23.8	0.001
	1199	SKIN EXFOLIATION	1673	54	24.3	2.2	1.000	16.4	3.3	0.001
	1634	NAIL DISCOLOURATION	429	91	6.2	14.6	0.927	4.2	21.6	0.001
0200		Musculo-skeletal system disorders	50,667	2387	735.9	3.2	0.011	497.7	4.8	0.001
	0063	ARTHRALGIA	8201	416	119.1	3.5	0.813	80.6	5.2	0.001
	0073	MYALGIA	24,679	1908	358.4	5.3	0.003	242.4	7.9	0.001
0410		Central & peripheral nervous system disorders	233,135	2081	3386.1	0.6	1.000	2290.1	0.9	1.000
	0148	SENSORY DISTURBANCE	2473	767	35.9	21.4	0.003	24.3	31.6	0.001
	1313	NEUROPATHY PERIPHERAL	5634	230	81.8	2.8	0.986	55.3	4.2	0.001
	1532	LOWER MOTOR NEURONE LESION	117	12	1.7	7.1	1.000	1.1	10.4	0.001
0431		Vision disorders	17,634	186	256.1	0.7	1.000	173.2	1.1	1.000
	1049	LACRIMATION ABNORMAL	647	116	9.4	12.3	0.879	6.4	18.3	0.001
	1462	EPIPHORA	151	22	2.2	10.0	1.000	1.5	14.8	0.001
0433		Special senses other, disorders	4692	563	68.1	8.3	0.121	46.1	12.2	0.001
	0267	TASTE PERVERSION	4195	555	60.9	9.1	0.103	41.2	13.5	0.001
0500		Psychiatric disorders	129,819	1261	1885.5	0.7	1.000	1275.2	1.0	1.000
	0165	ANOREXIA	36,109	690	524.5	1.3	1.000	354.7	1.9	0.001
0600		Gastro-intestinal system disorders	636,320	6813	9242.1	0.7	1.000	6250.7	1.1	1.000
	0204	CONSTIPATION	45,356	991	658.8	1.5	0.988	445.5	2.2	0.001
	0269	ANUS DISORDER	321	17	4.7	3.6	1.000	3.2	5.4	0.005
	0298	HAEMORRHOIDS	1442	45	20.9	2.1	1.000	14.2	3.2	0.005
	0321	PROCTITIS	91	24	1.3	18.2	0.999	0.9	26.8	0.001
	0327	STOMATITIS	10,870	256	157.9	1.6	1.000	106.8	2.4	0.001
	1014	HAEMORRHAGE RECTUM	655	33	9.5	3.5	1.000	6.4	5.1	0.001
	1083	GINGIVITIS	1353	133	19.7	6.8	0.949	13.3	10.0	0.001
	1351	MUCOSITIS NOS	4978	170	72.3	2.4	0.999	48.9	3.5	0.001
	1376	TOOTH ACHE	1032	42	15.0	2.8	1.000	10.1	4.1	0.001
0700		Liver and biliary system disorders	52,619	643	764.3	0.8	1.000	516.9	1.2	1.000
	0360	SGPT INCREASED	12,811	341	186.1	1.8	0.998	125.8	2.7	0.001
0800		Metabolic and nutritional disorders	68,669	714	997.4	0.7	1.000	674.6	1.1	1.000
	0381	HYPERCHOLESTEROLAEMIA	1982	139	28.8	4.8	0.978	19.5	7.1	0.001
	0387	HYPOCALCAEMIA	2433	112	35.3	3.2	0.998	23.9	4.7	0.001
1040		Vascular (extracardiac) disorders	13,671	255	198.6	1.3	1.000	134.3	1.9	0.001
	0207	FLUSHING	5334	216	77.5	2.8	0.986	52.4	4.1	0.001
	1413	ERYTHROMELALGIA	81	27	1.2	22.9	0.998	0.8	33.9	0.001
1100		Respiratory system disorders	137,936	1644	2003.4	0.8	1.000	1355.0	1.2	1.000
	0523	PHARYNGITIS	18,340	361	266.4	1.4	1.000	180.2	2.0	0.003
1210		Red blood cell disorders	30,116	1675	437.4	3.8	0.030	295.8	5.7	0.001
	0544	ANAEMIA	25,889	1668	376.0	4.4	0.011	254.3	6.6	0.001
1220		White cell and RES disorders	92,531	3969	1344.0	3.0	0.002	909.0	4.4	0.001
	0570	AGRANULOCYTOSIS	8089	375	117.5	3.2	0.899	79.5	4.7	0.001
	0572	GRANULOCYTOPENIA	58,735	2474	853.1	2.9	0.028	577.0	4.3	0.001
	0908	LEUCOPENIA	20,456	1091	297.1	3.7	0.167	200.9	5.4	0.001
1300		Urinary system disorders	49,509	301	719.1	0.4	1.000	486.3	0.6	1.000
	0621	RENAL PAIN	466	40	6.8	5.9	1.000	4.6	8.7	0.001
1420		Reproductive disorders, female	10,695	283	155.3	1.8	1.000	105.1	2.7	0.001
	0636	AMENORRHOEA	800	151	11.6	13.0	0.761	7.9	19.2	0.001
	0669	VAGINITIS	463	23	6.7	3.4	1.000	4.5	5.1	0.001
	1839	BREAST PAIN	500	38	7.3	5.2	1.000	4.9	7.7	0.001
1810		Body as a whole—general disorders	220,690	4995	3205.4	1.6	0.334	2167.9	2.3	0.001
	0401	OEDEMA PERIPHERAL	9444	607	137.2	4.4	0.398	92.8	6.5	0.001
	0716	ASTHENIA	24,301	456	353.0	1.3	1.000	238.7	1.9	0.006
	0717	BACK PAIN	9781	209	142.1	1.5	1.000	96.1	2.2	0.003
	0718	CHEST PAIN	25,856	928	375.5	2.5	0.681	254.0	3.7	0.001
	0724	FATIGUE	14,561	515	211.5	2.4	0.933	143.0	3.6	0.001
	1705	TEMPERATURE CHANGED SENSATION	9204	822	133.7	6.1	0.079	90.4	9.1	0.001
	1765	PALMAR-PLANTAR ERYTHRODYSAESTHESIA	7415	517	107.7	4.8	0.441	72.8	7.1	0.001
	2101	PAIN AXILLARY	159	20	2.3	8.7	1.000	1.6	12.8	0.001
1820		Application site disorders	25,336	150	368.0	0.4	1.000	248.9	0.6	1.000
	0058	INJECTION SITE REACTION	3385	106	49.2	2.2	1.000	33.3	3.2	0.001
2000		Secondary terms—events	12,322	92	179.0	0.5	1.000	121.0	0.8	0.001
	1813	SURGICAL SITE REACTION	290	68	4.2	16.1	0.964	2.8	23.9	0.001

## Conclusion and discussion

This study sought to reveal how the tree-based scan statistic developed by Kulldorff et al.^26^ can be extended for the zero-inflated count data. To consider a large number of zero cells, we proposed the TreeScan-ZIP method, which integrates a zero-inflated Poisson model into the TreeScan-Poisson method. Herein, a simulation study was conducted with different settings for the relative risk and the number of true zero leaves and true signal leaves. Based on the findings of the simulation study, the TreeScan-ZIP method performed better than the TreeScan-Poisson method in terms of power, sensitivity, and PPV, especially when the proportion of true zeros was high. The real data examples also supported the simulation results. The TreeScan-Poisson method may have missed many signals that were detected by the TreeScan-ZIP method in datasets with a large number of true zeros. If the TreeScan-ZIP method detects too many false positive signals, it may increase confusion in further investigation and utilize unnecessary energy. However, even the known AEs were not detected by the TreeScan-Poisson method. Although we do not know whether all signals detected by the TreeScan-ZIP method were true, it is safer to over-detect than to miss any signal in drug safety surveillance.

The data used were extracted from spontaneous reporting systems, which is a limitation. As spontaneous reporting systems are based on self-reporting by people, such as consumers and healthcare professionals, underreporting or overreporting of AEs may easily occur. For example, only the number of cases reported can be known. Thus, whether the same AE occurred multiple times in the same person cannot be known. Cases of overreporting may thus lead to bias in the analysis.

In this study, the TreeScan-ZIP method and TreeScan-Poisson method identified signals of AEs for a particular drug, and could identify drugs that are more frequently reported to be related to a particular AE. Cuts were made either above or below nodes in this study; however, more elaborate cuts, such as the combinational cuts proposed by Kulldorff et al.^7^ can also be made. In this study, we used a two-level structure; however, structures with more than two levels or other spontaneous reporting system data with more delicate levels can be employed. Further studies could use a zero-inflated double Poisson or zero-inflated negative binomial model to accommodate large numbers of true zeros and overdispersion^43^. When a priory level of AE definition cannot be determined in the tree structure and the data have a large number of zeros, the proposed tree-based scan statistic can serve as a very useful method for detecting signals in the post-market drug safety surveillance.

## Data availability

The KARES database is provided via the Korea Institute of Drug Safety and Risk management webpage. (https://open.drugsafe.or.kr/original/invitation.jsp) upon request.
